# Progressive Medicine

**Published:** 1902-03

**Authors:** 


Progressive Medicine. A Quarterly Digest of Advances, Discoveries and Improvements in the Medical and Surgical Sciences. Edited by Hobart Amory Hare, M. D., Professor of Therapeutics and Materia Medica in the Jefferson Medical College of Philadelphia, etc. Assisted by H. R. M. Landis, M. D., Assistant Physician to the Out-Patient Department of the Jefferson Medical College Hospital. Volume IV. December, 1900. Lea Brothers & Co., Philadelphia and New York.

This volume embraces seven exhaustive articles. The first is by

Max Einhorn, M. D., Professor of Diseases of the Stomach and Alimentary Tract in the Post-Graduate Medical School, New York, on the "Diseases of the Digestive Tract and Allied Organs, the Liver, Pancreas and Peritoneum." The second article is devoted to "Genito-Urinary Diseases and Syphilis," by Wm. T. Belfield, M. D., Professor of Surgery in the Chicago Polyclinic, etc. Dr. J. C. Bloodgood contributes a most excellent article on the "Fractures, Dislocations, Amputations, Surgery of the Extremities, and Orthropedics." Dr. John Rose Bradford, Professor of Materia Medica and Therapeutics in the University College, London, etc., writes on "Diseases of the Kidneys." Albert P. Brubaker, M. D., Adjunct Professor of Physiology and Hygiene in the Jefferson Medical College, Philadelphia. Dr. Henry B. Baker, of the Michigan State Board of Health, Lansing, contributes the article on "Hygiene."

The above articles are exhaustive, and review the field of the world for the year. T. J. B.

